# scDR: Predicting Drug Response at Single-Cell Resolution

**DOI:** 10.3390/genes14020268

**Published:** 2023-01-19

**Authors:** Wanyue Lei, Mengqin Yuan, Min Long, Tao Zhang, Yu-e Huang, Haizhou Liu, Wei Jiang

**Affiliations:** 1 Department of Biomedical Engineering, Nanjing University of Aeronautics and Astronautics, Nanjing 211106, China; 2 College of Automation, Nanjing University of Aeronautics and Astronautics, Nanjing 211106, China

**Keywords:** drug resistance, drug response, cancer, heterogeneity, scRNA-seq

## Abstract

Heterogeneity exists inter- and intratumorally, which might lead to different drug responses. Therefore, it is extremely important to clarify the drug response at single-cell resolution. Here, we propose a precise single-cell drug response (scDR) prediction method for single-cell RNA sequencing (scRNA-seq) data. We calculated a drug-response score (*DRS*) for each cell by integrating drug-response genes (DRGs) and gene expression in scRNA-seq data. Then, scDR was validated through internal and external transcriptomics data from bulk RNA-seq and scRNA-seq of cell lines or patient tissues. In addition, scDR could be used to predict prognoses for BLCA, PAAD, and STAD tumor samples. Next, comparison with the existing method using 53,502 cells from 198 cancer cell lines showed the higher accuracy of scDR. Finally, we identified an intrinsic resistant cell subgroup in melanoma, and explored the possible mechanisms, such as cell cycle activation, by applying scDR to time series scRNA-seq data of dabrafenib treatment. Altogether, scDR was a credible method for drug response prediction at single-cell resolution, and helpful in drug resistant mechanism exploration.

## 1. Introduction

Drug resistance, mainly caused by tumor heterogeneity (TH), is one of the huge challenges for effective treatment for cancers. The complex genetic contexts of tumor cells, exhibiting inter- and intra-TH, account for different drug responses. Targeted therapy is an optimal therapeutic management of tumors. Some anti-cancer drugs have been developed and applied in clinical cancer therapies by targeting genes such as EGFR, KRAS, etc. [1]. However, the barrier for targeted therapy is that tumor cells might gradually become resistant to the drug as treatment proceeds.

In contrast to tissue samples, cell lines, from the original tumor, are still considered to be the main vector for exploring molecular and pharmacological characteristics because of their properties of being relatively easy to culture, observe, and carrying rich genetic information about drug response [2]. Several projects focusing on pharmacogenomics based on cancer cell lines have systematically provided large-scale resources of drug response and gene expression profiles such as the Cancer Cell Line Encyclopedia (CCLE) [3], Genomics of Drug Sensitivity in Cancer (GDSC) [4] and Cancer Therapeutics Response Portal (CTRP) [5], which offered an opportunity to link drug response and genomic features. Numerous studies have systematically identified drug-resistance-related genes [6,7] and non-coding RNAs [8,9]. However, the poor reproducibility of these single markers limits their extended applications. In contrast to single markers, previous studies have demonstrated that the multi-gene markers or gene set have more stable performance [10,11]. Therefore, identifying drug-resistance-related multi-gene signature will help to determine the drug response of tumor cells.

With the development of single-cell RNA sequencing (scRNA-seq) technology, gene expression profile enables resolution at the single-cell level [12]. Over the past ten years, numerous studies have been performed to determine TH using scRNA-seq data [13,14]. For example, Wu et al. found that tumors from different patients with advanced non-small cell lung cancer displayed extensive inter-TH in cellular composition, chromosomal structure, developmental trajectory, intercellular signaling network, and phenotype dominance [13]. Zhou et al. presented a single-cell atlas to explore intra-TH, and provided potential therapeutic targets for osteosarcoma [14]. Recently, scRNA-seq data of 198 cell lines from 22 cancer types have been provided to describe the landscape of heterogeneity within diverse cancer cell lines [15]. It is of great significance to predict drug response at single-cell resolution, which is useful for overcoming drug resistance and leading to effective treatment and diagnosis. However, a few studies have focused on drug response prediction based on scRNA-seq data [16,17]. Wang et al. developed a knowledgebase, ceDR, reporting the computational inference of cellular drug responses for hundreds of cell types [16]. Beyondcell calculates an enrichment score in a collection of drug signatures, delineating therapeutic clusters within cellular populations [17]. Currently, a lack of systematically effective validation of the two methods limits their applications. In this study, we developed a precise single-cell drug response (scDR) prediction method through integrating scRNA-seq data of multiple drug-response genes. Herein, we will describe the generation, application, and comparative usefulness of the scDR method. Based on our rigorous analysis of scDR, we believe that it introduces a robust and essential new tool to the drug response prediction at single-cell resolution.

## 2. Materials and Methods

### 2.1. Datasets Collection and Pre-Processing

Five cohorts were used in this study, including one discovery cohort, three validation cohorts, and one application cohort (Table 1). The Discovery Cohort was used to screen drug-response genes (DRGs). The gene expression profiles for 56,202 Ensembl genes and 1019 cancer cell lines were downloaded from CCLE (https://sites.broadinstitute.org/ccle, accessed on 17 September 2021) [3]. Genome annotation file was downloaded from the Ensembl website (https://www.ensembl.org, accessed on 19 September 2021). The protein-coding genes in the genome annotation file intersected with the genes in CCLE were extracted, and 18,782 genes were retained for further analysis. The drug response data, measured by the area under percent-viability curves (AUCs) for 481 drugs across 664 cancer cell lines, were obtained from CTRP Version 2 (http://portals.broadinstitute.org/ctrp, accessed on 17 September 2021) [5]. Detailed information of cohorts used in this study is summarized in Table 1.

Three validation cohorts were used for evaluating the accuracy and stability of scDR. Validation Cohort I and II were the bulk RNA-seq data in cell lines and tissues, respectively. Validation Cohort III was the scRNA-seq data in cell lines. In Validation Cohort I, we downloaded the gene expression profiles for 1018 cell lines from GDSC (https://www.cancerrxgene.org, accessed on 20 September 2021) [4]. The 424 cell lines with matched drug response data in CTRP were retained. In Validation Cohort II, we downloaded gene expression profiles and clinical information for 33 cancer types from TCGA (https://portal.gdc.cancer.gov, accessed on 23 September 2021) [18]. Samples with drug response information were retained, including clinical progressive disease (PD) and complete response (CR). For each drug-cancer pair, samples with PD and CR were regarded as resistant and sensitive, respectively. In this study, three drug-cancer pairs (Fluorouracil–STAD, Gemcitabine–PAAD, and Gemcitabine–BLCA) were retained for further analysis according to the following criteria: first, the DRGs for this drug could be obtained in the Discovery Cohort; second, the numbers of PD and CR samples for this drug were both greater than 15. In Validation Cohort III, we downloaded the scRNA-seq data of 198 cell lines analyzed by Kinker et al. [15]. The R package Seurat [19] was used to perform scRNA-seq analysis. Gene expression profiles were imported into Seurat for quality control and downstream analysis. Low-quality cells and genes (< 200 genes/cell, > 20% mitochondrial genes/cell, and < 3 cells/gene) were excluded. Raw counts were normalized using the NormalizeData function. The detailed information is summarized in Table 1 and Table 2.

scDR was applied to time series scRNA-seq data of melanoma (Application Cohort). The expression profiles of the SKMEL28 cell line, treated with dabrafenib for 0, 1, 2, and 3 days, were downloaded from Gene Expression Omnibus (GEO, GSE162045). The R package Seurat, following the same preprocessing as Validation Cohort III, was used to obtain normalized data. Highly variable genes were recognized for principal component analysis (PCA). Subsequently, the top 10 PCs were used as inputs to t-distributed Stochastic Neighbor Embedding (t-SNE) [20]. The FindCluster function using the resolution of 0.12 was applied to analyze cell clusters. The FindMarkers function was used to calculate differentially expressed genes (|log_2_*FC*| > 1, adjusted *p*-value < 0.05) between cell clusters.

### 2.2. scDR Method

#### 2.2.1. Step 1. Identifying Resistant and Sensitive Cell Lines

The first step of the scDR was to obtain resistant and sensitive cell lines. First, for a drug, *d*, we separated the cell lines as resistant (*R*) and sensitive (*S*) using AUC values in CTRP, detailed as follows:

(1) $$\left\{\begin{array}{c} if\;\mathrm{AUC}\;>mean+0.8\times sd,R\\ if\;\mathrm{AUC}\;<\;mean-0.8\times sd,S \end{array}\right.$$

where, *mean* and *sd* represent the average and standard deviation of AUC values of all cell lines, respectively.

#### 2.2.2. Step 2. Predicting Drug-Response Genes (DRGs)

Then, we performed differential expression analysis between *R* and *S* cell lines. We calculated the log-transformed fold change (log_2_*FC*) of each gene based on gene expression level as follows:

(2) $$\log_{2}FC={\;\log}_{2}\frac{rowMeans(exp_{R})}{rowMeans(exp_{S})}$$

where, *rowMeans(exp_R_)* and *rowMeans(exp_S_)* represent the average gene expression in the *R* and *S* cell lines for one drug, respectively. We took the top 200 upregulated and the top 200 downregulated genes as drug-response genes (DRG_d_) of drug *d.* According to the above process, we predicted the DRGs and the corresponding log_2_*FC* values of 481 drugs in CTRP (Table S1).

#### 2.2.3. Step 3. Calculating Drug-Response Scores (*DRSs*)

In order to calculate the *DRS*, the gene expression profiles were first normalized. Then, the expression level of gene *i* in sample *m* was transformed by *Zscore* as follows:

(3) $$Zscore_{im}=\frac{exp_{im}-Mean\left(exp_{i}\right)}{Sd\left(exp_{i}\right)}$$

where *exp_im_* represents the expression level of gene *i* in sample *m*; *Mean*(*exp_i_*) represents the average of gene expression levels in all samples; and *Sd*(*exp_i_*) represents the standard deviation of gene expression levels in all samples. Finally, *DRS_dm_* (*DRS* for sample *m* and drug *d*) can be calculated as follows:

(4) $$DRS_{dm}=\frac{\sum_{i=1}^{q}\left(\log_{2}FC_{id}\times Zscore_{im}\right)}{q}$$

where log_2_*FC_id_* represents the log_2_*FC* value of gene *i* for drug *d*, which is trained in Discovery Cohort; *q* is the number of intersected genes of DRGs and gene expression profiles.

### 2.3. Validation of scDR Method

Firstly, we used scDR to predict *DRSs* in the Discovery Cohort and compared the difference of the predicted *DRSs* between *R* and *S* cell lines corresponding to one drug using one-sided Wilcoxon tests. The significant difference (*p*-value < 0.05) was regarded as an accurate prediction. After obtaining the significance level for all 481 drugs, we calculated the accuracy of scDR. Then, we also performed internal and external validation to verify the reliability of scDR. For internal validation, threefold cross-validation was performed. The gene expression profiles of *R* and *S* cell lines corresponding to one drug were randomly divided into three equal groups separately. One resistant group and one sensitive group were combined as the test set. The remaining cell lines were considered as the training set. We used the training set to re-obtain DRGs and the related log_2_*FC* values, and re-calculated *DRSs* for samples in the test set. In order to compare the results from different tests, we scaled the *DRS_dm_* into [0,1] in each test. For each drug, we performed 100 permutations and obtained 900 tests to verify the accuracy of scDR. We evaluated the difference in the predicted *DRSs* between resistant and sensitive cell lines corresponding to one drug using one-sided Wilcoxon test. Similarly, the significant difference (*p*-value < 0.05) was regarded as an accurate prediction and calculated accuracy in all 481 drugs. In addition, we verified scDR with external datasets, including the bulk RNA-seq of cell lines (Validation Cohort I) and tissue samples (Validation Cohort II), as well as the scRNA-seq of cell lines (Validation Cohort III). Similarly, the significance of *DRSs* difference between resistant and sensitive samples was used to evaluate the accuracy of scDR. Finally, scDR was also compared with the existing method, Beyondcell [17], using the weighted probabilistic concordance index (WPCI) [21]. WPCI is a modification of the concordance index (c-index), which can be used to compare the consistency between true and the predicted drug responses (Supplementary Methods). Compared with the c-index, WPCI further considers the variance within the true drug responses (AUC). Here, WPCI was used to evaluate the performance of drug response prediction methods.

### 2.4. Survival Analysis

To explore whether *DRSs* could serve as prognostic marker or not, we performed survival analysis for STAD, PAAD, and BLCA (Validation Cohort II). In addition, we downloaded the expression and clinical data of SKCM in TCGA to perform survival analysis for upregulated genes in d0-R cluster in the Application Cohort. Survival analysis was performed using the survfit function in the R survival package (v3.4-0); Kaplan–Meier (KM) survival curves were plotted using the ggsurvplot function in the R package survminer (v0.4.9).

### 2.5. Pseudotime Analysis

We performed pseudotime analysis for melanoma cells to determine the process of evolution under dabrafenib treatment. Pseudotime analysis was carried out using the Monocle toolkit [22]. Subsequently, the differentialGeneTest function was adopted to determine differential expressed genes between the clusters. Dimensionality reduction analysis of the cells was carried out using the DDRTree approach and the reduceDimension function based on the differential expressed genes. Through the orderCells function, the cells along the quasi-chronological trajectory were sorted and visualized.

### 2.6. Gene Set Enrichment Analysis

To validate that the d0-R cluster in the Application Cohort was the intrinsic resistant cell subgroup, we performed single sample gene set enrichment analysis (ssGSEA) for d0-R and d0-S cells. Tumor microenvironment (TME)-associated signatures, which were cancer-malignant and drug resistance-related, were downloaded from the previous collection in IOBR [23]. ssGSEA was performed to calculate the score of signatures using the R package GSVA (v1.36.3) [24].

To detect the key potential mechanisms associated with resistance-related markers, gene set enrichment analysis for the Kyoto Encyclopedia of Genes and Genomes (KEGG) [25] and Gene Ontology (GO) [26] was performed for d0-R up-regulated genes by Metascape (https://metascape.org, accessed on 2 November 2021) [27]. The parameters were set as follows: Min Enrichment = 1.5, *p*-value Cutoff = 0.05, Min Overlap = 3.

### 2.7. Statistical Analysis

One-sided Wilcoxon tests were used to compare the *DRSs* and ssGSEA scores between different groups. A *p*-value < 0.05 was considered statistically significant.

## 3. Results

### 3.1. Development of scDR

**Figure 1:**
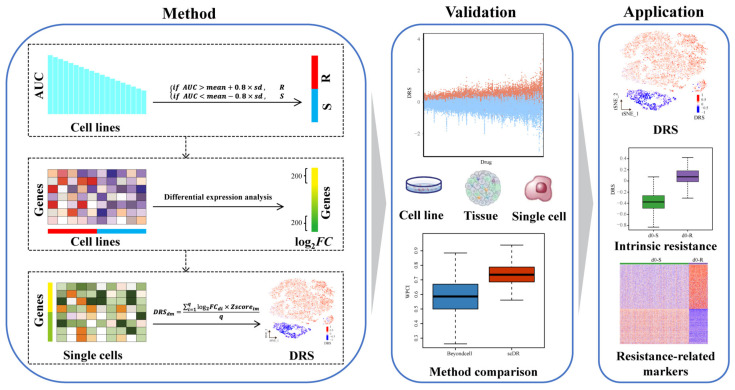
Workflow of scDR.

The scDR framework involves three major steps: (1) identifying resistant and sensitive cell lines; (2) predicting drug-response genes (DRGs); and (3) calculating drug-response scores (*DRSs*) (Figure 1, more details in Section 2 Materials and Methods). Briefly, we inferred the DRGs of 481 drugs based on CCLE gene expression profiles and CTRP drug response data. We compared the overlaps of DRGs and GO terms which were significantly enriched in DRGs (adjusted *p*-value < 0.05) for different drugs (Supplementary Methods, Tables S1 and S2). The results showed that the DRGs and GO terms of most drugs were unique, and only a few drugs shared more DRGs and GO terms, such as gefitinib, erlotinib, lapatinib, afatinib, neratinib, and vandetanib, all of which are inhibitor of EGFR. (Figure S1). Next, we calculated the drug response score (*DRS*) for each cell using the scRNA-seq data of the DRGs. Then, we validated the accuracy and the stability of scDR using three data cohorts, including the bulk RNA-seq of cell lines and patient tissues, as well as the scRNA-seq of cell lines. In addition, scDR was applied to time series scRNA-seq data of melanoma cell lines during dabrafenib treatment. We identified an intrinsic resistant cell subgroup in the untreated cell lines, probably maintained by the upregulation of cell cycle genes such as RAN and TUBA1B.

### 3.2. Internal Validation Based on Cell Lines

**Figure 2:**
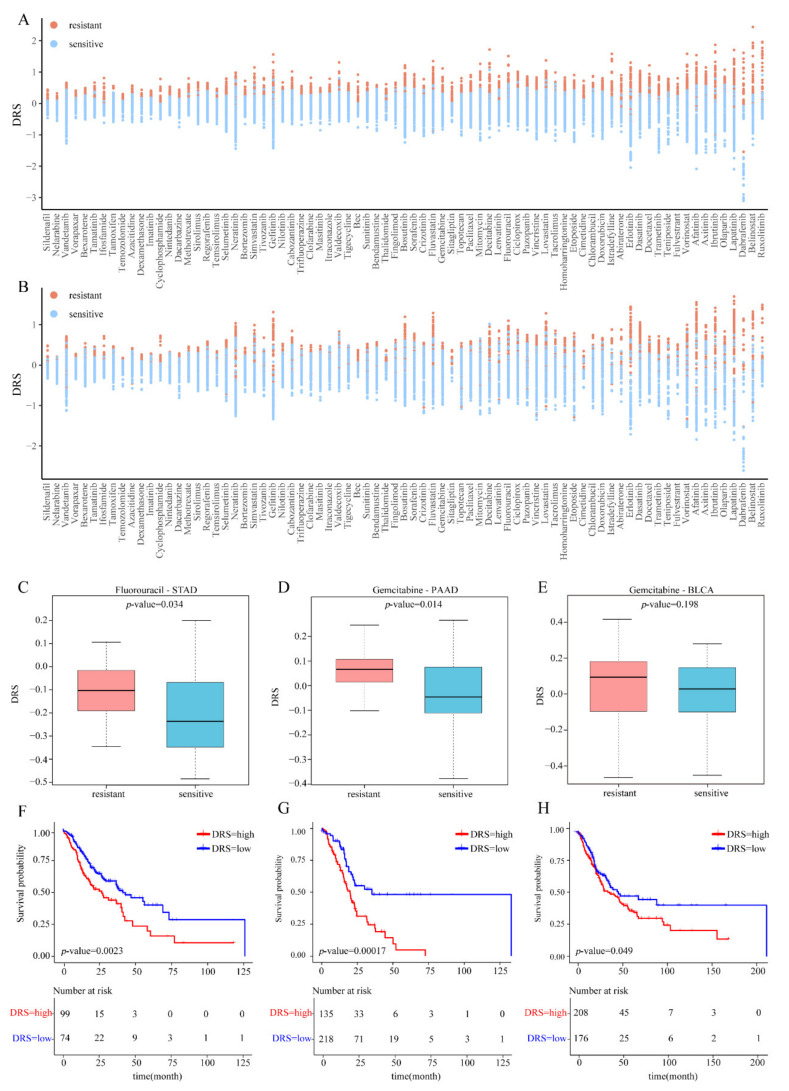
Validation of scDR. (**A**). The dot plot of *DRSs* for 77 FDA-approved drugs using the Discovery Cohort. The red dots represent resistant cell lines, and the blue dots represent sensitive cell lines. The *x*-axis represents 77 FDA-approved drugs. The *y*-axis shows the *DRS*. (**B**). External validation using Validation Cohort I. The dot plot of *DRSs* for 77 FDA-approved drugs. The red dots represent resistant cell lines, and the blue dots represent sensitive cell lines. The *x*-axis represents 77 FDA-approved drugs. The *y*-axis shows the *DRS*. (**C**–**H**). Validation using Validation Cohort II. Box plot of *DRSs* for resistant and sensitive samples in three drug-cancer pairs (**C**–**E**). Kaplan–Meier curves for patients with different cancers (STAD, PAAD, and BLCA) according to the mean value of *DRSs* (**F**–**H**).

We identified the DRGs and calculated the *DRSs* in the Discovery Cohort, which include the expression profiles of 1019 cell lines in CCLE and the drug response data (AUC) of 481 drugs in CTRP. We evaluated the difference in the predicted *DRSs* between the resistant and sensitive cell lines corresponding to each drug using one-sided Wilcoxon test. The significant difference (*p*-value < 0.05) was regarded as an accurate prediction. The results showed that the accuracy was 100% (481 of 481) in the whole Discovery Cohort (Figure S2). The *DRS*s of the resistant and sensitive cell lines for 77 FDA-approved drugs was shown in Figure 2A. In addition, we evaluated the accuracy of scDR using threefold cross-validation in the Discovery Cohort. We performed differential expression analysis in the training set and calculated the *DRSs* of the resistant and sensitive cell lines in the test sets (details in Section 2 “Materials and Methods”). The accuracy was 91.27% (439 of 481, Figure S3). These results suggested that scDR was an accurate method to predict drug response.

### 3.3. External Validation Using Bulk RNA-Seq Data of Cell Lines

In addition to internal validation, we also performed external validation for scDR accuracy and stability using three Validation Cohorts, including the bulk RNA-seq data of cell lines (Validation Cohort I) and tissues (Validation Cohort II), as well as scRNA-seq data of cell lines (Validation Cohort III). The Validation Cohort I contained the gene expression profiles and the matched drug response data of 424 cell lines. We calculated the *DRS*s of 481 drugs in the 424 cell lines. The differential *DRS* analysis on the resistant and sensitive cell lines showed that the *DRSs* of resistant cell lines were significantly (*p*-value < 0.05) higher than those of the sensitive cell lines for 479 of 481 (99.58%) drugs (Figure S4). Especially for 77 FDA-approved drugs, 100% of drugs exhibited significant differences in *DRSs* between the resistant and sensitive cell lines (Figure 2B). The performance indicated the accuracy and stability of scDR in predicting drug responses for cell lines.

### 3.4. External Validation Using Bulk RNA-Seq Data of TCGA Patients

To further explore the effectiveness of scDR for clinical patients, we applied scDR to three drug-cancer pairs (Fluorouracil–STAD, Gemcitabine–PAAD, and Gemcitabine–BLCA) in TCGA (Validation Cohort II). The patients were assigned to two groups based on the different drug responses: resistant (patients showing “Clinical Progressive Disease”) and sensitive (patients showing “Complete Response”). We calculated the *DRSs* of three datasets. Higher *DRSs* were observed in the resistant samples for all three drug-cancer pairs (Figure 2C–E). These results showed that the scDR could effectively predict the drug response for clinical patients. In addition, we aimed to determine whether the *DRSs* could predict survival time for cancer patients. We divided the samples into high- and low-*DRS* groups according to the average of *DRSs*. Survival analysis revealed that high-*DRS* patients had shorter survival times. Significant differences (*p*-value < 0.05) were observed in all three cancers (Figure 2F–H). Overall, the *DRSs* can be used to predict drug response and survival risk for clinical patients.

### 3.5. Drug Response Prediction and Method Comparison in the scRNA-Seq Data

**Figure 3:**
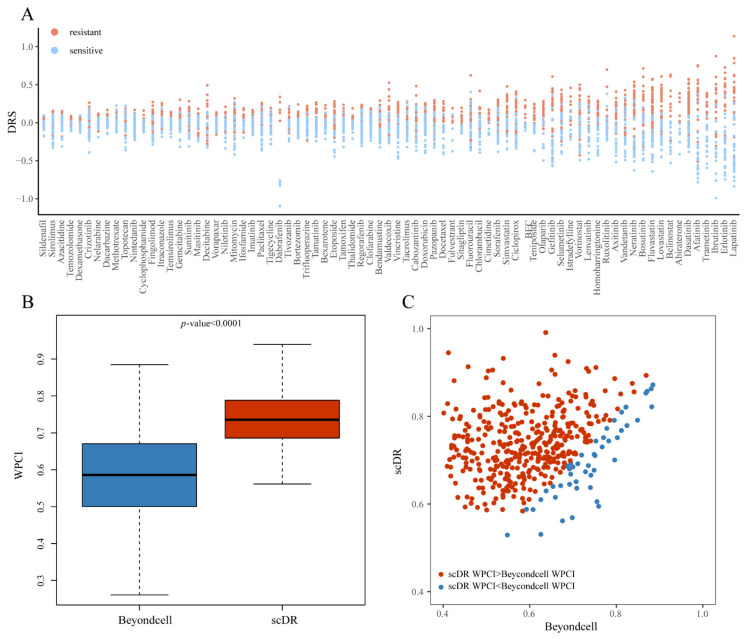
Validation and method comparison using scRNA-seq data. (**A**). The dot plot of *mDRS* predicted by scDR for 77 FDA-approved drugs. The *x*-axis represents 77 FDA-approved drugs. The *y*-axis shows the *DRS*. The red dots represent the resistant cell lines. The blue dots represent the sensitive cell lines. (**B**). The box plot of WPCI for scDR and Beyondcell. The *y*-axis shows the WPCI. (**C**). The dot plot of WPCI. Each dot represents one drug.

In the above analysis, we validated that the scDR method was effective for drug response prediction in bulk RNA-seq data for both cell lines and patient tissues. Here, we further verified scDR in the scRNA-seq data. The single-cell expression profiles of 53,502 cells of 198 cell lines covering 22 cancers were obtained from a prior study [15]. We used scDR to predict the *DRS* of each cell for 481 drugs. There was no true information about the drug response at single-cell level; therefore, we calculated the mean *DRSs* values (*mDRS*) of cells in each cell line and performed a comparison between resistant and sensitive cell lines. Thus, the differences in *mDRS* showed that, for 460 of 481 (95.63%) drugs, the resistant cell lines had significantly (*p*-value < 0.05) higher *mDRS* values than the sensitive cell lines (Figure S5). Especially for the FDA-approved drugs, 74 of 77 (96.1%) of them exhibited significant differences (Figure 3A). Furthermore, we compared the proposed scDR with the existing method Beyondcell [17]. Here, we used the WPCI to evaluate the prediction performance by considering the rank concordance between the true drug response (AUC) and the predicted *DRS*. A higher WPCI value meant a higher accuracy of the method (details in Section 2 Materials and Methods). In general, the WPCI of scDR was significantly (*p*-value < 0.0001) higher than that of Beyondcell (Figure 3B). Specifically, for 426 of 475 (89.68%) drugs, WPCI of scDR was higher than that of Beyondcell (Figure 3C). For 71 of 76 (93.42%) FDA-approved drugs, the WPCI of scDR was higher than that of Beyondcell (Figure S6). These results indicated that scDR could accurately predict drug responses at single-cell resolution.

### 3.6. Applying scDR to Identify Intrinsic Resistant Cell Subgroups in Melanoma Based on scRNA-Seq Data

**Figure 4:**
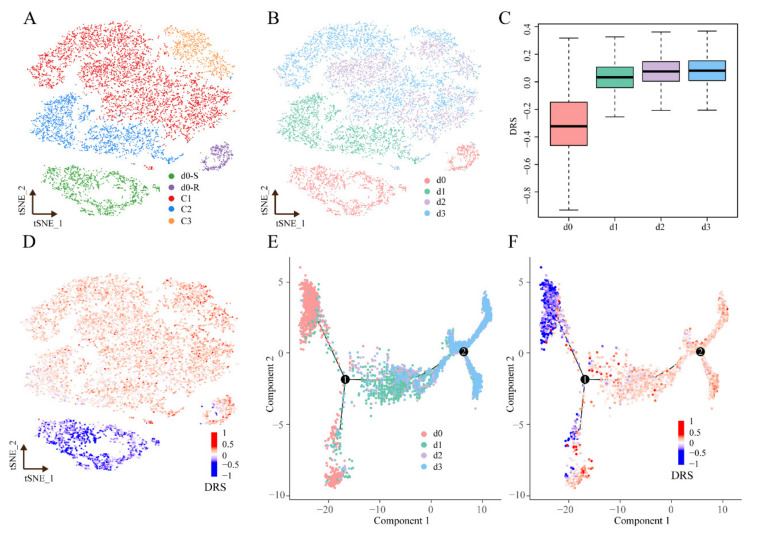
Determination of intrinsic resistant cell subgroups in melanoma using scRNA-seq data. (**A**) The t-SNE plot for cell clusters. (**B**) The t-SNE plot colored by dabrafenib treatment time. (**C**) The box plot of *DRSs* of cells treated with dabrafenib for 0, 1, 2, and 3 days. (**D**) The t-SNE plot colored by *DRS*s. (**E**) Pseudotime cell trajectories colored by treatment time. (**F**) Pseudotime cell trajectories colored by *DRS*s.

We applied scDR to identify intrinsic resistant cell subgroups based on the scRNA-seq data of the melanoma cell line SKMEL28 treated with dabrafenib for 0, 1, 2, and 3 days (Application Cohort). We used the function “FindCluster” in the R package Seurat to perform clustering analysis. These cells were grouped into five clusters at the resolution of 0.12, which were named d0-R, d0-S, C1, C2, and C3 (Figure 4A). The cells without drug treatment (0 day) were divided into two clusters (Figure 4B). We found that the *DRS* increased as time proceeded (Figure 4C). This indicated that cells were becoming more and more resistant as drug treatment proceeded longer and longer, which is the usual characteristic of drug treatment. Cells treated with dabrafenib for 1, 2, and 3 days, all had high *DRS*s (Figure 4D). However, the two clusters in the cells without drug treatment (0 day) showed significant difference in *DRS*s (*p*-value < 0.0001) (Figure 4D and Figure S7A). The high-*DRS* cluster was named as d0-R, and the low-*DRS* cluster was named as d0-S. These results suggested that the heterogeneity existed in the untreated cells. Next, the monocle toolkit was used to reorder single cells into a pseudo-temporal timeline; the results clearly demonstrated the evolution trend from 0 to 3 days (Figure 4E). We observed that the *DRSs* increased with the pseudo-temporal timeline (Figure 4F), and there were two separated branches in 0 day for tumor cells (Figure S7B). These results indicated that the cells in d0-R were more likely to be intrinsic resistant.

**Figure 5:**
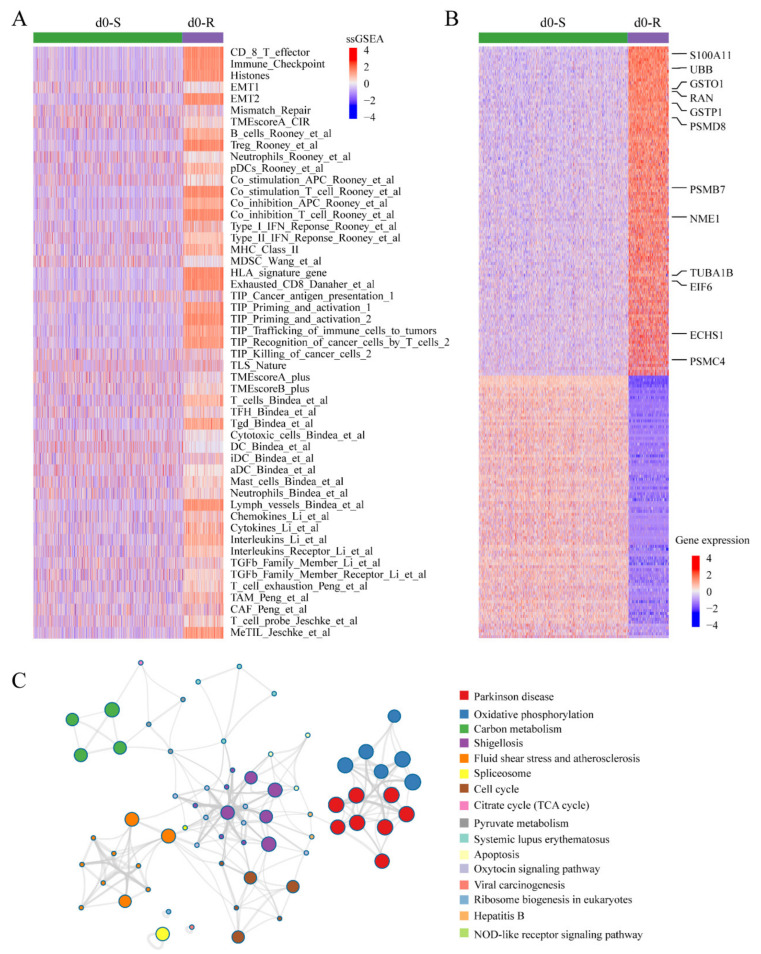
Functional analysis for d0-S and d0-R cells. (**A**) The heatmap shows the TME-associated signatures with significantly different ssGSEA scores between d0-R and d0-S cells. (**B**) The heatmap shows differentially expressed genes between d0-R and d0-S cells. (**C**) Enrichment map of KEGG pathways for the upregulated genes in cluster d0-R. The node size represents the number of genes in a pathway. The edge width represents the similarities between two pathways.

To further demonstrate that d0-R was an intrinsic resistant cluster, we performed ssGSEA for TME-associated signatures, which were collected as cancer-malignant and drug resistance-related signatures in a previous study [23]. In 51 TME-associated signatures, d0-R exhibited significantly (*p*-value < 0.05) higher ssGSEA scores (Figure 5A), such as EMT, mismatch repair and immune checkpoint, which has been demonstrated to play important roles in drug resistance [28,29,30]. These results further verified the drug-resistant characteristics of d0-R cells and the accuracy of scDR. Furthermore, we performed gene differential expression analysis between d0-R and d0-S using the FindMarkers function in Seurat. The genes with *|*log_2_*FC|* > 1 and adjusted *p*-value < 0.05 were identified as significantly differentially expressed. The results showed 113 significantly upregulated and 90 significantly downregulated genes in d0-R (Figure 5B), of which some genes are reportedly associated with drug resistance such as the cell-cycle-related genes RAN and TUBA1B [31,32,33]. To further demonstrate whether the upregulated genes in d0-R could be prognostic markers, we performed survival analysis for these genes and found that 12 genes were significantly (*p*-value < 0.05) prognosis-related including RAN and TUBA1B (Figure S8). In addition, we explored the biological function of the up-regulated genes in d0-R. The enrichment analysis showed that some drug resistance-related KEGG pathways and GO functions were significant (*p*-value < 0.05), such as the “cell cycle” and “apoptosis” pathways [34,35,36] (Figure 5C and Figure S9). These results showed that scDR was useful for determining the heterogeneity of the drug response and exploring the potential mechanisms of drug resistance.

## 4. Discussion

For tumor precision therapy, to accurately distinguish resistant cells from sensitive cells is necessary. Although amounts of scRNA-seq data arise to push on the exploration of tumor heterogeneity, a computational method of predicting drug response and identifying resistant cells for scRNA-seq data is still lacking. Here, we present scDR, a precise single-cell drug response prediction method for scRNA-seq data. From bulk RNA-seq data, drug response signatures were obtained and then transferred to predict drug responses in scRNA-seq. scDR could accurately predict drug responses for both bulk RNA-seq and scRNA-seq data, as well as for both cell lines and clinical patient tissues. In addition, compared with the existing method, scDR achieved higher accuracy. Finally, we showed a case that scDR could aid in intrinsic resistant cell identification and dissection of biological mechanisms. Thus, scDR could help accurate resistant cells identification and the determination of biological mechanisms. Similarly, we also used scDR to predict the drug response in an additional scRNA-seq data for breast cancer cells (GSE131135, detailed in Supplementary Methods), and found that the *DRSs* of drug-resistant cells was higher than that of sensitive cells (Figure S10). Thus, users could apply our method to accurately evaluate the drug response at single-cell resolution and identify resistant subgroups of tumor cells, which is of great value for precision medicine.

The effectiveness of scDR has been validated in drug response prediction; however, it still has potential for improvement. First, drug response signature was simply obtained by differential expression analysis. Other signature identification method might lead to improvements, such as scDEAL [37]. Second, current methods all overlooked relevant cell–cell information for scRNA-seq data. It has been demonstrated that considering cell–cell networks could enable distinguishing between different groups of cells, such as SCAVENGE [38], ikarus [39], Scissor [40], and scDEAL [37]. Third, it might supply more biological knowledge to consider drug response signatures from respective cell functions or pathways. Although these fields need more exploration, scDR should be used in drug response predictions and provide novel insights for cancer therapy.

## Figures and Tables

**Table 1:** Summary of data cohorts used in this study.

Data Set	Data Source	Cancer	Data Type	Samples
Discovery Cohort	CTRP	Pan-Cancer	Drug response	664
	CCLE	Pan-Cancer	RNA-seq	1019
Validation Cohort I	GDSC	Pan-Cancer	RNA-seq	424
Validation Cohort II	TCGA	STAD	RNA-seq	353
		PAAD	RNA-seq	173
		BLCA	RNA-seq	385
Validation Cohort III	Kinker et al.	Pan-Cancer	scRNA-seq	53,502
Application Cohort	GSE162045	Melanoma	scRNA-seq	10,050

**Table 2:** Data summary of three drug-cancer pairs used in Validation Cohort II.

Cancer	Drug	PD	CR
STAD	Fluorouracil	16	17
PAAD	Gemcitabine	28	19
BLCA	Gemcitabine	19	28

## Data Availability

All the datasets used in this study were from public resources and have been described in the Section 2 Materials and Methods. The code used in this study is available from the corresponding authors upon reasonable request.

## Supplementary Materials

The following supporting information can be downloaded at: https://www.mdpi.com/article/10.3390/genes14020268/s1. File S1: Supplementary Methods [21,41]. Table S1. DRGs of 481 drugs. Table S2. DRGs-enriched Gene Ontology (GO) terms of 77 FDA-approved drugs. Figure S1. DRGs similarity of 77 FDA-approved drugs. (A) Heatmap of the Jaccard similarity index of DRGs for different drugs. Jaccard similarity index of the up-regulated and down-regulated DRGs were calculated, and the average of them was regarded as final Jaccard similarity index of DRGs. (B) Heatmap of the Jaccard similarity index of Gene Ontology (GO) terms for different drugs. GO enrichment analyses of the up-regulated and down-regulated DRGs were performed using the R package clusterProfiler with the adjusted *p*-value cutoff of 0.05. Jaccard similarity index of the up-regulated and down-regulated DRGs-enriched GO terms were calculated, and the average of them was regarded as final Jaccard similarity index of GO terms. Figure S2. Internal validation using the Discovery Cohort. (A) The dot plot of *DRSs* for 481 drugs. The *x*-axis represents 481 drugs; the *y*-axis represents *DRSs*. The red dots represent the resistant cell lines; the blue dots represent the sensitive cell lines. (B) The distribution of drugs according to *p*-values of one-sided Wilcoxon tests (calculated by differential *DRS* analysis between drug-resistant and drug-sensitive cell lines). The *x*-axis represents the *p*-value, and the *y*-axis represents the number of drugs. Figure S3. Distribution of drugs according to *p*-values of one-sided Wilcoxon tests (calculated by differential *DRS* analysis between drug-resistant and drug-sensitive cell lines) in threefold cross-validation in the Discovery Cohort. The *x*-axis represents the *p*-value, and the *y*-axis represents the number of drugs. Figure S4. External validation in the Validation Cohort I. (A) The dot plot of *DRSs* for 481 drugs. The *x*-axis represents 481 drugs; the *y*-axis represents *DRS*. The red dots represent the resistant cell lines, the blue dots represent the sensitive cell lines. (B) The distribution of drugs according to *p*-values of one-sided Wilcoxon tests (calculated by differential *DRS* analysis between drug-resistant and drug-sensitive cell lines). The *x*-axis represents the *p*-value, and the *y*-axis represents the number of drugs. Figure S5. External validation in the Validation Cohort III. (A) The dot plot of *mDRSs* for 481 drugs. The *x*-axis represents 481 drugs, and the *y*-axis represents *mDRS*. The red dots represent drug-resistant cell lines. The blue dots represent drug-sensitive cell lines. (B) The distribution of drug according to *p*-values of one-sided Wilcoxon tests (calculated by differential *DRS* analysis between resistant and sensitive cell lines). The *x*-axis represents the *p*-value, and the *y*-axis represents the number of drugs. Figure S6. Comparison of accuracy between scDR and Beyondcell. The *x*-axis represents the FDA-approved drugs. The *y*-axis represents the weighted probabilistic c-index (WPCI). Figure S7. (A) Box plot of *DRS*s for d0-S and d0-R cells. (B) Pseudotime cell trajectories colored by cell clusters. Figure S8. Kaplan–Meier curves for SKCM patients according to mean values of gene expression. Resistance-related genes significantly related to survival are shown. Figure S9. Enrichment map of Gene Ontology (GO) for the upregulated genes in cluster d0-R. The node size represents the number of genes in a GO term. The edge width represents the similarities between GO terms. Figure S10. Box plot of *DRSs* for SUM159DMSO (sensitive cells) and SUM159RDMSO (resistant cells) cell lines.
