# Supplementary material for: scDR: Predicting Drug Response at Single-Cell Resolution
Source: Genes (Basel). 2023 Jan 19;14(2):268. doi: 10.3390/genes14020268 (PMC9957092; doi:10.3390/genes14020268)
Supplement: Supplementary file 1 [file genes-14-00268-s001.zip › genes-2076205-supplementary.pdf]

scDR: predicting drug response at single-cell resolution

Wanyue Lei[1†], Mengqin Yuan[1†], Min Long[1], Tao Zhang[1], Yu-e Huang[1], Haizhou Liu[2*] and Wei Jiang[1*]

Supplementary Methods

Similarity of DRGs and their enriched functions of different drugs

We inferred drug-response genes (DRGs) of 481 drugs based on CCLE gene expression profiles and CTRP drug response data (details in Materials and Methods). We used Jaccard similarity index to evaluate the similarity of DRGs and their enriched functions of different drugs. Take drug *a* and drug *b* as example, the Jaccard similarity index for the up-regulated DRGs can be calculated as follows:

$$J(a,b)_{U\_DRG} = \frac{|U\_DRG_a \cap U\_DRG_b|}{|U\_DRG_a \cup U\_DRG_b|} \quad (1)$$

where, U_DRG_a and U_DRG_b represent the up-regulated DRGs of drug *a* and *b*, respectively. Similarly, the Jaccard similarity index for the down-regulated DRGs can be calculated as follows:

$$J(a,b)_{D\_DRG} = \frac{|D\_DRG_a \cap D\_DRG_b|}{|D\_DRG_a \cup D\_DRG_b|} \quad (2)$$

where, D_DRG_a and D_DRG_b represent the down-regulated DRGs of drug *a* and *b*, respectively. The final Jaccard similarity index of DRGs can be calculated as follows:

$$J(a,b)_{DRG} = \frac{J(a,b)_{U\_DRG} + J(a,b)_{D\_DRG}}{2} \quad (3)$$

Next, gene ontology (GO) enrichment analysis for the up-regulated and down-regulated DRGs were performed using the R package clusterProfiler [1] with the adjusted *p*-value cutoff of 0.05. Then we compared the similarity of the DRG*s*-enriched functions. Take drug *a* and drug *b* as example, the Jaccard similarity index of the up-regulated DRGs-enriched GO terms can be calculated as follows:

$$J(a,b)_{U\_GO} = \frac{|U\_GO_a \cap U\_GO_b|}{|U\_GO_a \cup U\_GO_b|} \quad (4)$$

where, U_GO_a and U_GO_b represent the up-regulated DRGs-enriched GO terms of drug *a* and *b*, respectively. Similarly, the Jaccard similarity index of the down-regulated DRGs-enriched GO terms as follows:

$$J(a,b)_{D\_GO} = \frac{|D\_GO_a \cap D\_GO_b|}{|D\_GO_a \cup D\_GO_b|} \quad (5)$$

where, D_GO_a and D_GO_b represent the down-regulated DRGs-enriched GO terms of drug *a* and *b*, respectively. The final Jaccard similarity index of DRGs-enriched GO terms can be calculated as follows:

$$J(a,b)_{GO} = \frac{J(a,b)_{U\_GO} + J(a,b)_{D\_GO}}{2} \tag{6}$$

Weighted probabilistic concordance index (WPCI)

WPCI [2] was used to evaluate the performance of drug response prediction methods. $T = \{t_1, t_2, \dots, t_i, \dots, t_n\}$ represents the true drug response of *n* cell lines, and $P = \{p_1, p_2, \dots, p_i, \dots, p_n\}$ is the predicted drug response of *n* cell lines. t_i and p_i represent the true the predicted drug response of cell line *i*, respectively. The probabilistic concordance index (PCI) of the two lists *T* and *P* is calculated as follows:

$$PCI(T,P) = \frac{2}{n(n-1)} \sum_{i<j} f\left(t_i, t_j, p_i, p_j, \sigma_d\right) \tag{7}$$

$$f\left(t_i, t_j, p_i, p_j, \sigma_d\right) = \begin{cases} \frac{1}{2}\left(1 + \mathrm{erf}\left(\frac{t_i - t_j}{2\sigma_d}\right)\right), & if\left(p_i > p_j\right) \\ 0.5 & if\left(p_i = p_j\right) \\ \frac{1}{2}\left(1 + \mathrm{erf}\left(\frac{t_j - t_i}{2\sigma_d}\right)\right), & if\left(p_i < p_j\right) \end{cases} \tag{8}$$

$$\mathrm{erf}(x) = \frac{2}{\sqrt{\pi}} \int_0^x e^{-y^2}\, dy \tag{9}$$

where, σ_d is the standard deviation of *T*. The WPCI is calculated by scaling *PCI* as follows:

$$WPCI = \frac{PCI - PCI_{min}}{PCI_{max} - PCI_{min}} \tag{10}$$

PCI_{max} represents the maximum *PCI* when *T* and *P* have the same rank; PCI_{min} represents the minimum *PCI* when *T* and *P* have the absolutely reversing rank.

Drug response prediction in an additional scRNA-seq data of breast cancer

scDR was applied to an additional scRNA-seq data of breast cancer. The scRNA-seq

data of JQ1-treated and untreated SUM159 parental (SUM159DMSO) and resistant SUM159R (SUM159RDMSO) cells were downloaded from Gene Expression Omnibus (GEO, GSE131135). Gene expression profiles were then imported into the Seurat for quality control and downstream analysis. Low-quality cells and genes (< 200 genes/cell, > 20% mitochondrial genes/cell and < 3 cells/gene) were excluded. Raw counts were normalized using the NormalizeData function. After quality control, 751 SUM159DMSO cells and 1130 SUM159RDMSO cells remained. We used scDR to predict drug-response scores (*DRSs*) of these cells. Then, the differential analysis of *DRSs* between SUM159DMSO cells and SUM159RDMSO cells was performed using one-sided Wilcoxon test.

References

1. Wu, T., et al., *clusterProfiler 4.0: A universal enrichment tool for interpreting omics data.* Innovation (Camb), 2021. **2**(3): p. 100141.
2. Costello, J.C., et al., *A community effort to assess and improve drug sensitivity prediction algorithms.* Nat Biotechnol, 2014. **32**(12): p. 1202-12.

Supplementary Tables

Table S1. DRGs of 481 drugs.

Table S2. DRGs-enriched Gene Ontology (GO) terms of 77 FDA-approved drugs.

Supplementary Figures

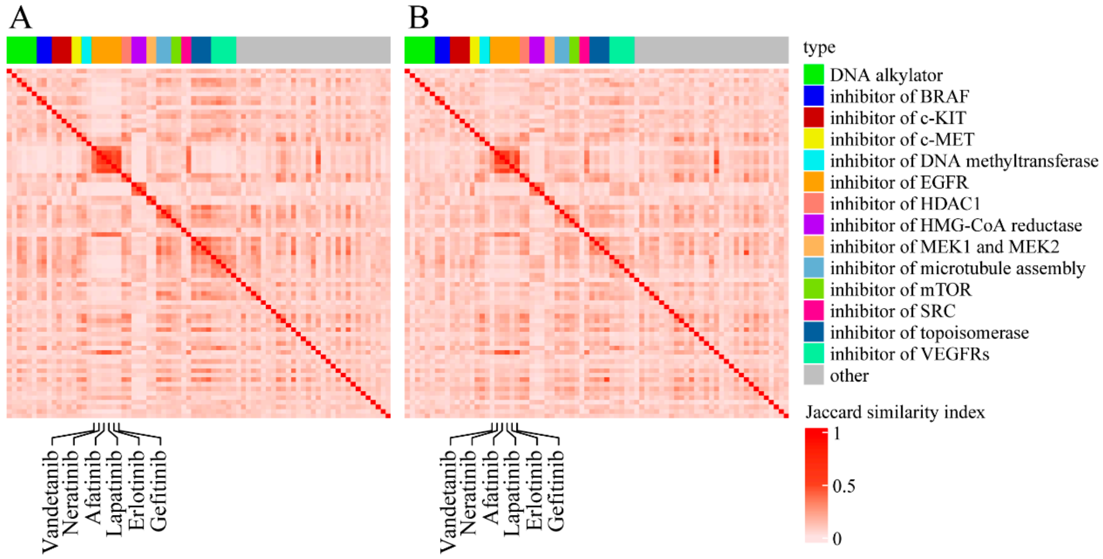

Figure S1. DRGs similarity of 77 FDA-approved drugs. (**A**) Heatmap of the Jaccard similarity index of DRGs for different drugs. Jaccard similarity index of the up-regulated and down-regulated DRGs were calculated, and the average of them was regarded as final Jaccard similarity index of DRGs. (**B**) Heatmap of the Jaccard similarity index of Gene Ontology (GO) terms for different drugs. GO enrichment analyses of the up-regulated and down-regulated DRGs were performed using the R package clusterProfiler with the adjusted *p*-value cutoff of 0.05. Jaccard similarity index of the up-regulated and down-regulated DRGs-enriched GO terms were calculated, and the average of them was regarded as final Jaccard similarity index of GO terms.

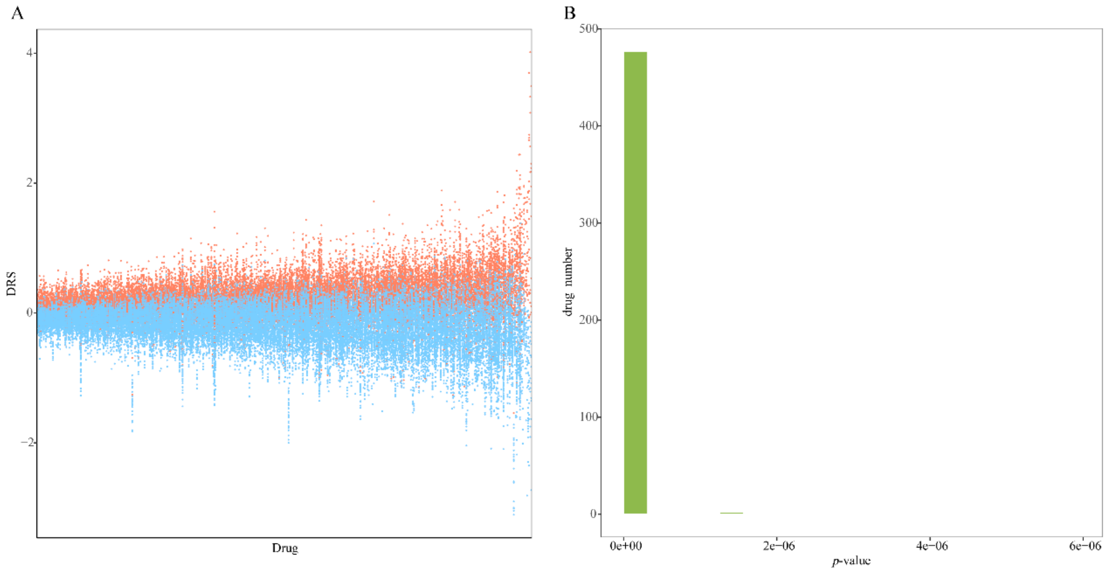

Figure S2. Internal validation using the Discovery Cohort. (**A**) The dot plot of *DRSs* for 481 drugs. The *x*-axis represents 481 drugs; the *y*-axis represents *DRSs*. The red dots represent the resistant cell lines; the blue dots represent the sensitive cell lines. (**B**) The distribution of drugs according to *p*-values of one-sided Wilcoxon tests (calculated by differential *DRS* analysis between drug-resistant and drug-sensitive cell lines). The *x*-axis represents the *p*-value, and the *y*-axis represents the number of drugs.

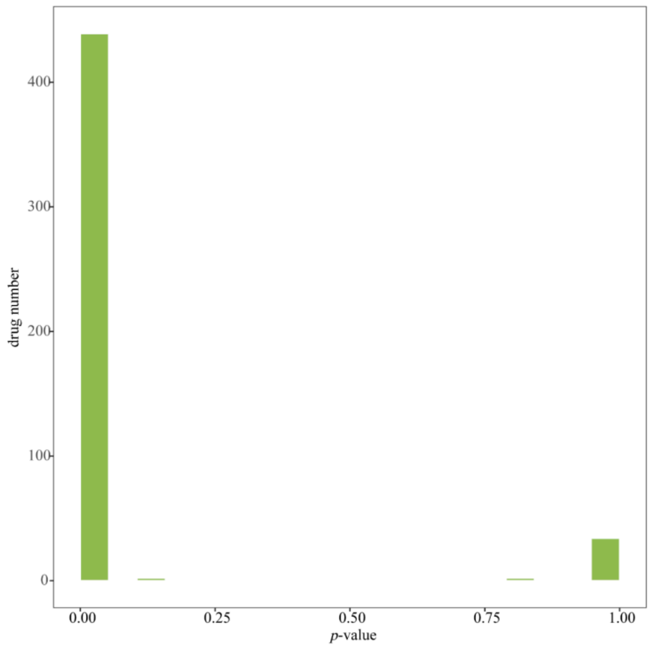

Figure S3. Distribution of drugs according to *p*-values of one-sided Wilcoxon tests (calculated by differential *DRS* analysis between drug-resistant and drug-sensitive cell lines) in threefold cross-validation in the Discovery Cohort. The *x*-axis represents the *p*-value, and the *y*-axis represents the number of drugs.

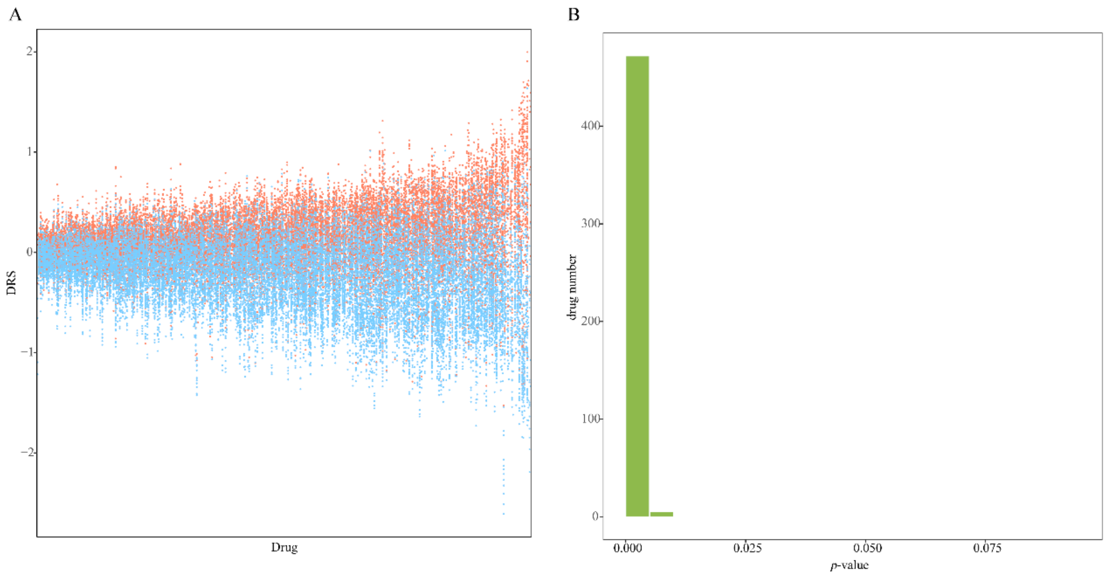

Figure S4. External validation in the Validation Cohort I. (**A**) The dot plot of *DRSs* for 481 drugs. The *x*-axis represents 481 drugs; the *y*-axis represents *DRS*. The red dots represent the resistant cell lines, the blue dots represent the sensitive cell lines. (**B**) The distribution of drugs according to *p*-values of one-sided Wilcoxon tests (calculated by differential *DRS* analysis between drug-resistant and drug-sensitive cell lines). The *x*-axis represents the *p*-value, and the *y*-axis represents the number of drugs.

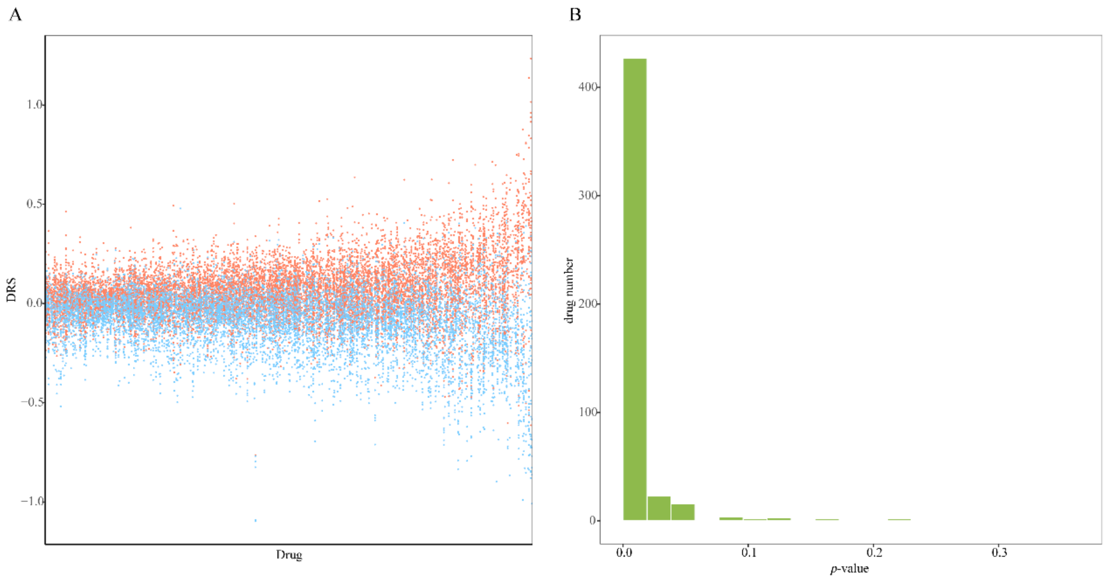

Figure S5. External validation in the Validation Cohort III. (**A**) The dot plot of *mDRSs* for 481 drugs. The *x*-axis represents 481 drugs, and the *y*-axis represents *mDRS*. The red dots represent drug-resistant cell lines. The blue dots represent drug-sensitive cell lines. (**B**) The distribution of drug according to *p*-values of one-sided Wilcoxon tests (calculated by differential *DRS* analysis between resistant and sensitive cell lines). The *x*-axis represents the *p*-value, and the *y*-axis represents the number of drugs.

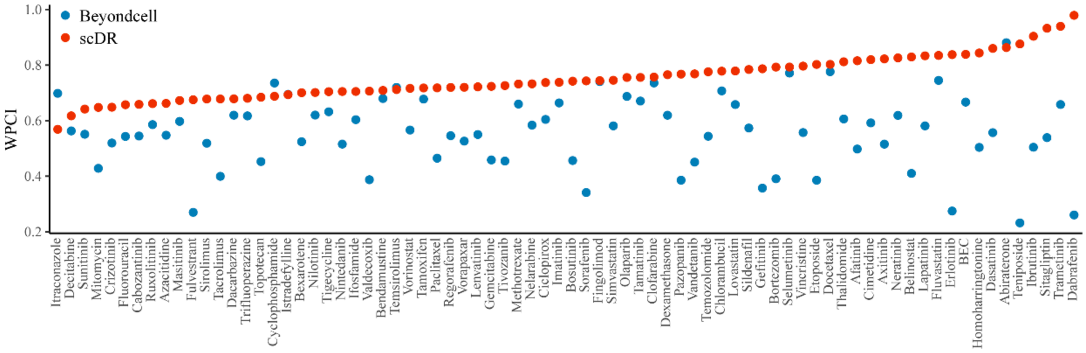

Figure S6. Comparison of accuracy between scDR and Beyondcell. The *x*-axis represents the FDA-approved drugs. The *y*-axis represents the weighted probabilistic c-index (WPCI).

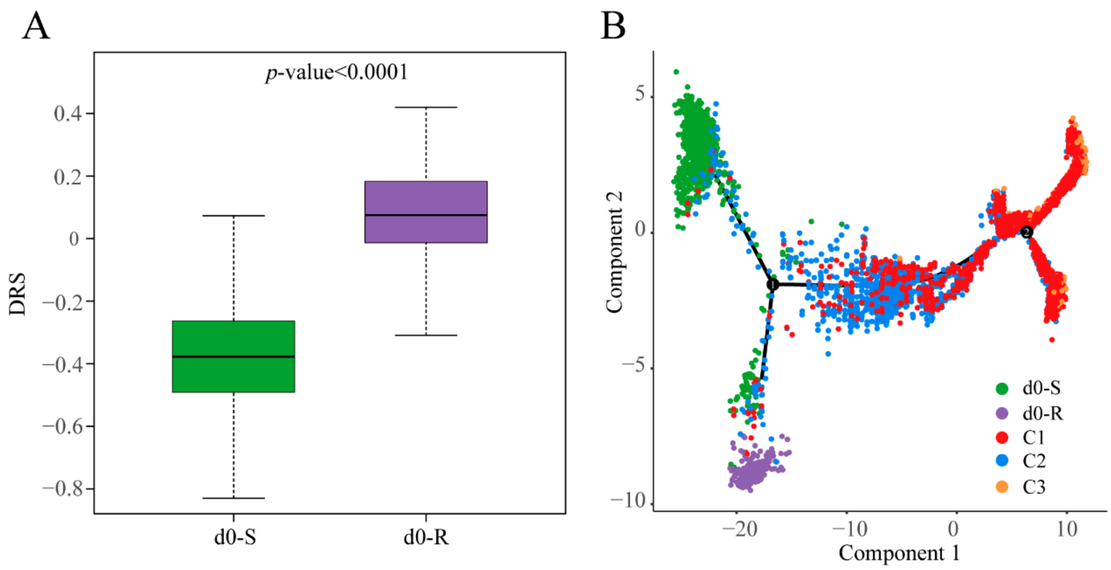

Figure S7. (**A**) Box plot of *DRS*s for d0-S and d0-R cells. (**B**) Pseudotime cell trajectories colored by cell clusters.

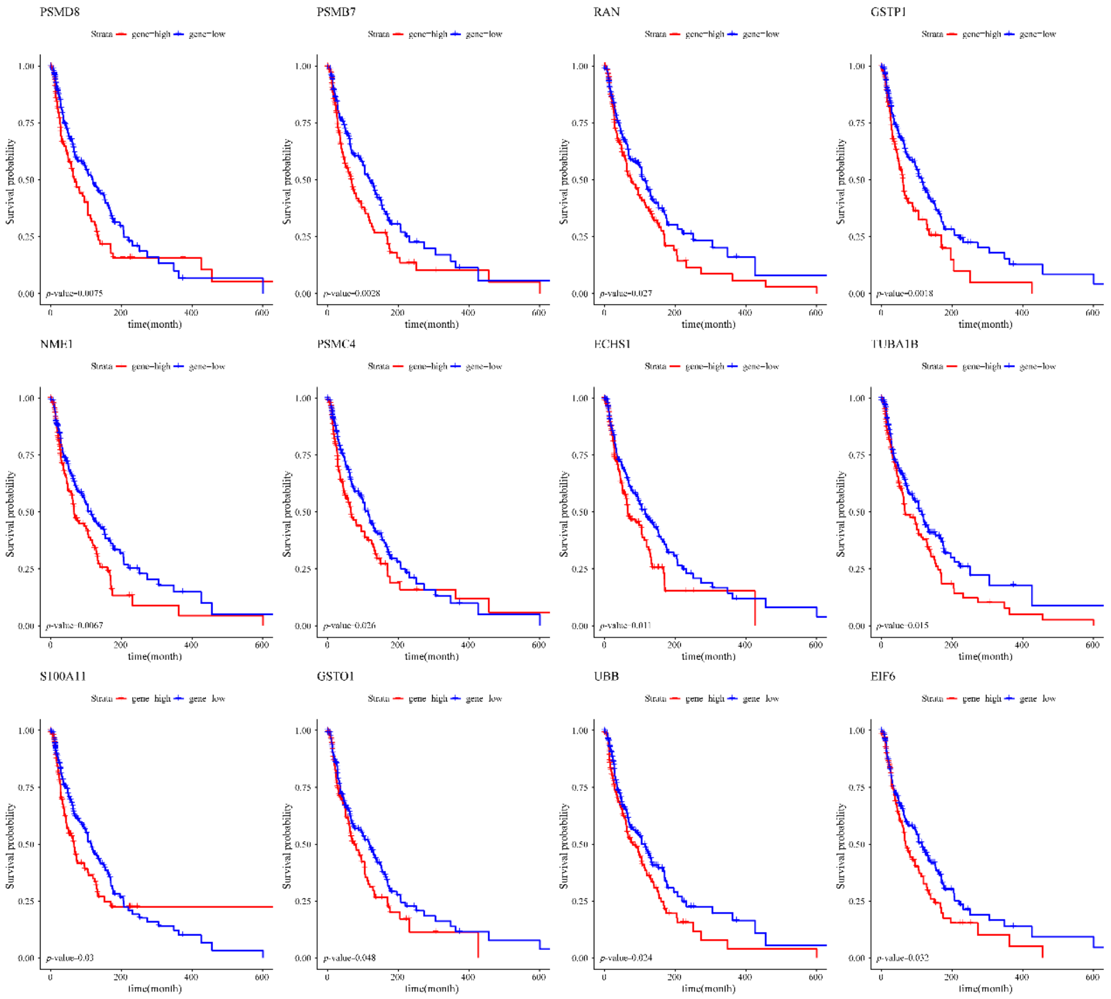

Figure S8. Kaplan-Meier curves for SKCM patients according to mean values of gene expression. Resistance-related genes significantly related to survival are shown.

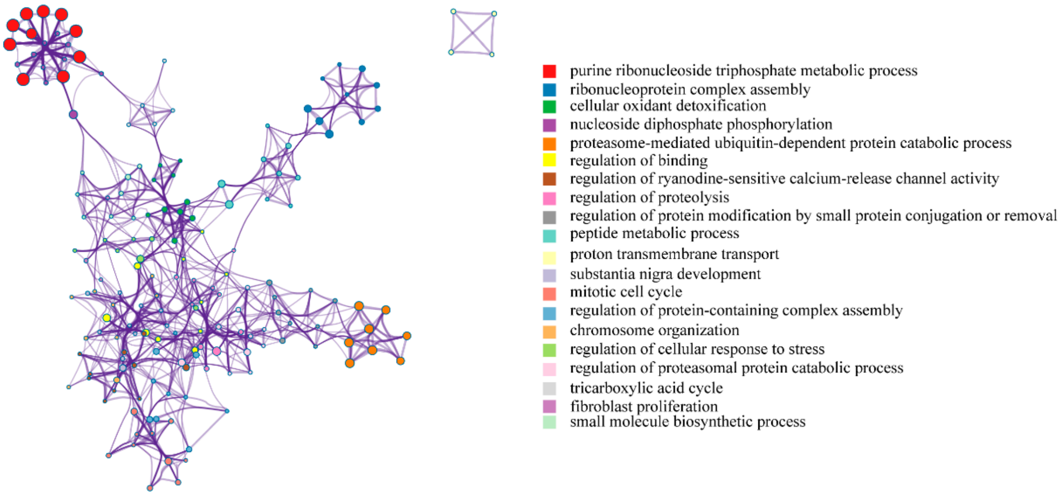

Figure S9. Enrichment map of Gene Ontology (GO) for the up-regulated genes in cluster d0-R. The node size represents the number of genes in a GO term. The edge width represents the similarities between GO terms.

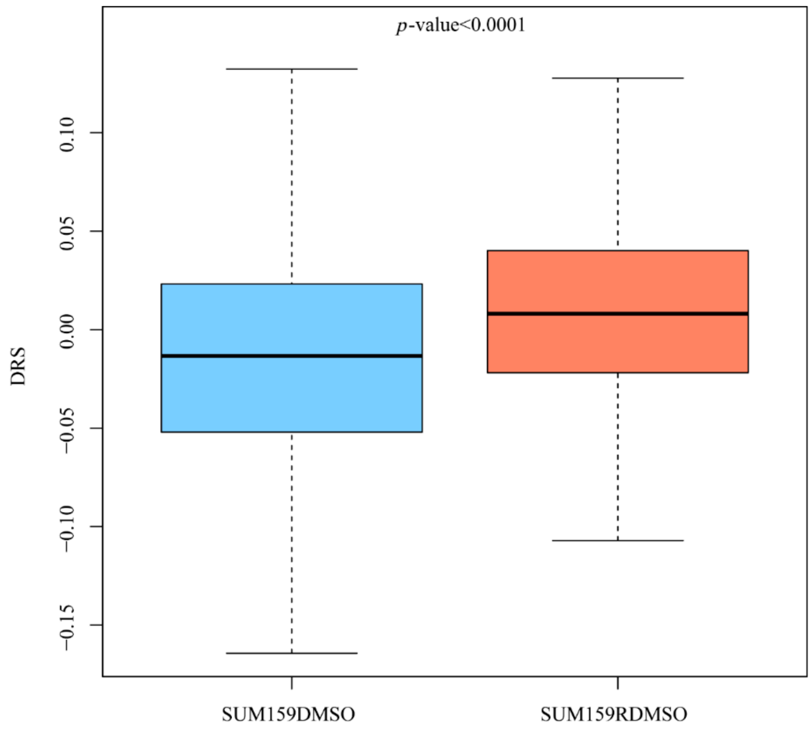

Figure S10. Box plot of *DRSs* for SUM159DMSO (sensitive cells) and SUM159RDMSO (resistant cells) cell lines.
